# Peritoneal Dialysis-Related Recurrent Rhodococcus corynebacterioides Peritonitis: A Case Report and Review of Literature

**DOI:** 10.7759/cureus.43423

**Published:** 2023-08-13

**Authors:** Maryam Saleem, Maryam Maqsood, Hassaan Iftikhar, Dhiren Haria, Hamza Arif

**Affiliations:** 1 Nephrology, Ohio Valley Nephrology Associates, Owensboro, USA; 2 Nephrology, Washington University School of Medicine, St. Louis, USA; 3 Internal Medicine, Waterbury Hospital, Waterbury, USA; 4 Internal Medicine, Mayo Hospital, Lahore, PAK; 5 Internal Medicine, King Edward Medical University, Lahore, PAK; 6 Internal Medicine, Saint Francis Medical Center, Trenton, USA

**Keywords:** peritoneal dialysis catheter, secondary peritonitis, rhodococcus corynebacterioides, end stage kidney disease (eskd), peritoneal dialysis complication, bacterial peritonitis

## Abstract

*Rhodococcus corynebacterioides* is a Gram-positive bacterium known to cause bacteremia and oligoarthritis. There have been only a few case reports in the literature that describe its association with peritoneal dialysis (PD)-related peritonitis. We report a case of recurrent peritonitis caused by *R. corynebacterioides*. The patient presented with abdominal pain, and PD fluid analysis was positive for infection, with cultures growing *R. corynebacterioides*. The patient was treated with multiple courses of intraperitoneal antibiotics due to recurrent episodes of PD-associated peritonitis from this bacterium, ultimately necessitating the removal of the PD catheter and the transition to hemodialysis.

## Introduction

Peritoneal dialysis (PD)-associated peritonitis is the most concerning complication of PD and is associated with significant morbidity and mortality [1]. Patients usually present with abdominal pain, and the PD fluid effluent cell count analysis shows an elevated WBC count greater than or equal to 100 cells/μL with at least 50% polymorphonuclear leukocytes. Cultures subsequently test positive for the causative organism. The most common etiologies include Gram-positive cocci, particularly coagulase-negative Staphylococcus, Streptococcus species, and Gram-negative bacilli [2, 3]. Peritonitis caused by Rhodococcus species, however, is rare and consequently less commonly described in the literature [4, 5]. *R. corynebacterioides*, formerly Nocardia corynebacterioides, is a partially acid-fast, Gram-positive rod with a slow growth rate [6]. Here, we present a case of relapsing peritonitis with culture-positive *R. corynebacterioides*. This is the first reported case of peritonitis associated with *R. corynebacterioides* in the United States.

## Case presentation

A 48-year-old male with a history of deep venous thrombosis due to factor V Leiden mutation, hereditary hemochromatosis, class I obesity, hypertension, and end-stage kidney disease (with an unclear cause) presented to the clinic. His renal biopsy showed non-specific chronic changes and global glomerulosclerosis, with no basement membrane abnormalities or evidence of immune complex-mediated diseases. He was on automated PD and came in with complaints of abdominal pain and cloudy effluent. PD fluid cell count and cultures were obtained, and the patient was started on empiric intraperitoneal (IP) ceftazidime for peritonitis. The cell count showed a total nucleated cell count (TNC) of 158 cells/uL, of which 89% were neutrophils. Three days later, the cultures grew gram-positive bacilli in an aerobic bottle, and direct cytospin grew variable organisms. At this point, IP ceftazidime was discontinued, and the patient was switched to IP vancomycin while waiting for cultures to be finalized. Due to technical difficulties in identifying the organism, an initial PD culture sample was sent to a specialty lab, which identified the organism three weeks later as *R. corynebacterioides*. The patient was then switched to oral ciprofloxacin for 10 days. The sensitivities of *R. corynebacterioides* were finalized, revealing sensitivity to ciprofloxacin. The patient’s abdominal pain persisted despite adherence to antibiotics; therefore, a repeat PD fluid analysis was obtained, which showed cloudy fluid, a TNC of 2492 cells/uL, and 61% neutrophils. Cultures remained negative. The patient was switched to IP amikacin (to which the organism was sensitive) and completed three weeks of this therapy. Repeat PD cultures two weeks after finishing antibiotics also remained negative, with complete resolution of symptoms.

One month after concluding antibiotics, he had another episode of abdominal pain and cloudy effluent. PD fluid cell count showed TNC of 316 cells/uL, of which 66% were neutrophils, requiring treatment with IP amikacin again for three weeks. Peritoneal fluid cultures later grew *R. corynebacterioides* this time as well. Two weeks after finishing antibiotics, repeat cell count and cultures remained without any evidence of infection, and clinically the patient had resolution of abdominal pain.

However, one month after the last dose of antibiotics, the patient had a similar presentation. PD cultures grew *R. corynebacterioides* once more. He was treated with IP gentamicin on this occasion. Due to recurrent peritonitis episodes, the PD catheter was removed, and the patient was transitioned to in-center hemodialysis (Table 1).

**Table 1:** Timeline of episodes of peritoneal dialysis-related peritonitis with treatment. PD: Peritoneal dialysis; TNC: Total neutrophil count; IP: Intraperitoneal.

Timeline	Symptoms	PD fluid cell count	PD fluid culture	Treatment and outcome
11/2022	Abdominal Pain; Cloudy Effluent.	TNC: 2697 cell/uL, 89% Neutrophils	No growth	IP vancomycin + IP cefepime for 2 weeks. Resolution of symptoms. Repeat PD fluid cultures one week after finishing antibiotics with no growth.
01/2023	Abdominal Pain; Cloudy Effluent.	TNC: 158 cell/uL, 89% Neutrophils	Rhodococcus corynebacterioides	IP vancomycin + IP ceftazidime for 7 days. Switched to oral ciprofloxacin when cultures and sensitivities resulted 3 weeks after the initial presentation. Abdominal pain persisted despite treatment with oral ciprofloxacin; switched to IP amikacin (susceptible).
03/2023	Surveillance Cultures	TNC <20 cell/uL,	No growth	
04/2023	Abdominal Pain; Cloudy Effluent.	TNC: 316 cell/uL, 66% Neutrophils	Rhodococcus corynebacterioides	IP amikacin for 3 weeks. Resolution of symptoms. Repeat cultures negative.
05/2023	Surveillance Cultures	TNC <20 cell/uL	No growth	
06/2023	Abdominal Pain; Cloudy Effluent.	TNC: 147 cell/uL, 94% Neutrophils	Rhodococcus corynebacterioides	IP amikacin for 2 weeks. PD catheter removed. Resolution of symptoms. Transitioned to in-center hemodialysis.

## Discussion

*Rhodococcus corynebacterioides*, previously known as *Nocardia corynebacterioides*, is a member of the family Nocardiaceae and the genus Rhodococcus. It is closely related to the genera Mycobacterium, Corynebacterium, and Nocardia [7]. This Gram-positive bacterium is widely spread across nature, primarily found in soil and water, where it plays a role in the biodegradation of a wide variety of substances. These include environmental pollutants, owing to their ability to use compounds such as carbohydrates and steroids as energy sources [8]. The genus Rhodococcus contains many other pathogenic strains, like R. equi, which is responsible for lung abscesses and pneumonia [9]. While *R. corynebacterioides* has been associated with neonatal bacteremia, oligoarthritis [10], sepsis, and bacteremia [11], much remains unknown about its association with PD. Literature on such cases is scarce, and the first reported case of peritonitis caused by any Rhodococcus species was published only recently, in 2021 [5]. The first instance of peritonitis caused by *R. corynebacterioides* was reported in 2022 [4].

*Rhodococcus corynebacterioides* is traditionally identified via gene sequencing and mass spectrometry, the former identifying it based on various genes such as genes involved in its metabolic pathways and virulence genes [12]. Traditional cultures, however, remain the gold standard since they provide us with valuable information about bacterial susceptibility and potential response to antibiotics. In contrast, techniques like mass spectrometry have shown a relatively higher false-negative rate [13]. In our patient's case, as soon as peritonitis was suspected, peritoneal fluid cultures were collected and sent to the lab. Meanwhile, empiric ceftazidime was started intraperitoneally in accordance with current guidelines. The cultures grew an aerobic Gram-positive bacillus; hence, the antibiotic was switched to IP vancomycin for better coverage. However, due to some difficulties associated with diagnosis, the exact organism was identified as *R. corynebacterioides* much later. Sensitivities were followed, and based on them, the patient was switched to oral ciprofloxacin. This closely reflected a similar pattern of sensitivities previously displayed by the bacterium in recorded cases so far [14]. Despite being sensitive to the treatment, the response remained subpar, and the symptoms persisted. Subsequent peritoneal fluid analysis showed elevated neutrophil counts, although the culture turned out negative. The patient was then switched to IP amikacin as the next best option, pending sensitivities. Following this change in treatment, the patient's symptoms began to improve. Although there is scarce data to support an optimal treatment duration, a similar case study had previously established a three-week duration, successfully treating the infection [4, 15]. We, therefore, followed suit. After this treatment period, the symptoms had completely resolved, and the cultures remained negative. Peritonitis, however, relapsed after a month with similar symptoms and cell counts, and the culture once again grew *R. corynebacterioides*. A noteworthy feature of the bacterium that can potentially explain its causing repeated relapses is its ability to form extensive biofilms. All Rhodococcus isolates have been shown to form biofilms, and hence they adhere to the surface of catheters [16]. This trait explains why relapses require catheter removal and appropriate antibiotic administration as the definitive treatment in most cases. Another course of IP amikacin was commenced to treat this relapse, and the duration and dose were the same as what was set in the previous episode. The catheter was left in place, as clinicians had planned for a conservative approach. Resolution of symptoms and culture negativity followed. Unfortunately, after a month of symptom resolution, another relapse occurred, and this time IP gentamicin was utilized. Also, keeping in view the repeated relapses, the catheter was removed, and the patient was switched to hemodialysis.

## Conclusions

Recurrent peritonitis has so far been a common occurrence when the pathogen in question is *R. corynebacterioides*. This case highlights the importance of early catheter removal to eliminate the risk of repeated relapses. While much remains to be explored about the pathogen, specifically concerning peritonitis, this case emphasizes key diagnostic modalities for its diagnosis. Culture is the most superior method, albeit time-consuming, with mass spectroscopy and gene sequencing serving as helpful additional guides. The safest treatment duration with antibiotics to achieve symptom resolution and a negative culture is closely aligned with previous similar cases. All episodes of peritonitis were treated adequately, as evidenced by negative culture and symptom resolution between episodes of relapsing peritonitis. However, the necessity of catheter removal and subsequent complete remission implies biofilm formation by this pathogen. Thus, physicians should keep this in mind when treating *R. corynebacterioides* peritonitis.

## References

[1] Li PK, Chow KM, Cho Y (2022). ISPD peritonitis guideline recommendations: 2022 update on prevention and treatment. Perit Dial Int.

[2] Perl J, Fuller DS, Bieber BA (2020). Peritoneal dialysis-related infection rates and outcomes: results from the Peritoneal Dialysis Outcomes and Practice Patterns Study (PDOPPS). Am J Kidney Dis.

[3] Whitty R, Bargman JM, Kiss A, Dresser L, Lui P (2017). Residual kidney function and peritoneal dialysis-associated peritonitis treatment outcomes. Clin J Am Soc Nephrol.

[4] Tanaka Y, Hirai D, Kawai Y, Ueda N, Takaori K, Koizumi M, Seta K (2023). Peritoneal dialysis-related peritonitis caused by Rhodococcus corynebacterioides. CEN Case Rep.

[5] Kang Y, Chen Y, Zhang Z, Shen H, Zhou W, Wu C (2021). A case of peritoneal dialysis-associated peritonitis caused by Rhodococcus kroppenstedtii. BMC Infect Dis.

[6] Majidzadeh M, Fatahi-Bafghi M (2018). Current taxonomy of Rhodococcus species and their role in infections. Eur J Clin Microbiol Infect Dis.

[7] Yassin AF, Schaal KP (2005). Reclassification of Nocardia corynebacterioides Serrano et al. 1972 (Approved Lists 1980) as Rhodococcus corynebacterioides comb. nov. Int J Syst Evol Microbiol.

[8] McLeod MP, Warren RL, Hsiao WW (2006). The complete genome of Rhodococcus sp. RHA1 provides insights into a catabolic powerhouse. Proc Natl Acad Sci USA.

[9] Yamshchikov AV, Schuetz A, Lyon GM (2010). Rhodococcus equi infection. Lancet Infect Dis.

[10] Khalil N, Corker L, Powell EA, Mortensen JE (2019). Neonatal bacteremia and oligoarthritis caused by Rhodococcus corynebacterioides/Rhodococcus kroppenstedtii. Diagn Microbiol Infect Dis.

[11] Kitamura Y, Sawabe E, Ohkusu K, Tojo N, Tohda S (2012). First report of sepsis caused by Rhodococcus corynebacterioides in a patient with myelodysplastic syndrome. J Clin Microbiol.

[12] Gürtler V, Mayall BC, Seviour R (2004). Can whole genome analysis refine the taxonomy of the genus Rhodococcus?. FEMS Microbiol Rev.

[13] Chang YT, Wang HC, Wang MC (2014). Rapid identification of bacteria and Candida pathogens in peritoneal dialysis effluent from patients with peritoneal dialysis-related peritonitis by use of multilocus PCR coupled with electrospray ionization mass spectrometry. J Clin Microbiol.

[14] Tang S, Lo CY, Lo WK, Chan TM (1997). Optimal treatment regimen for CAPD peritonitis caused by Rhodococcus species. Nephrol Dial Transplant.

[15] Szeto CC, Chow KM, Chung KY, Kwan BC, Leung CB, Li PK (2005). The clinical course of peritoneal dialysis-related peritonitis caused by Corynebacterium species. Nephrol Dial Transplant.

[16] Al Akhrass F, Al Wohoush I, Chaftari AM (2012). Rhodococcus bacteremia in cancer patients is mostly catheter related and associated with biofilm formation. PLoS One.

